# 3D Multiple Sound Source Localization by Proposed T-Shaped Circular Distributed Microphone Arrays in Combination with GEVD and Adaptive GCC-PHAT/ML Algorithms

**DOI:** 10.3390/s22031011

**Published:** 2022-01-28

**Authors:** Ali Dehghan Firoozabadi, Pablo Irarrazaval, Pablo Adasme, David Zabala-Blanco, Pablo Palacios Játiva, Cesar Azurdia-Meza

**Affiliations:** 1 Department of Electricity, Universidad Tecnológica Metropolitana, Av. José Pedro Alessandri 1242, Santiago 7800002, Chile; 2 Electrical Engineering Department, Pontificia Universidad Católica de Chile, Santiago 7820436, Chile; pim@uc.cl; 3 Biomedical Imaging Center, Pontificia Universidad Católica de Chile, Santiago 7820436, Chile; 4 Institute for Biological and Medical Engineering, Pontificia Universidad Católica de Chile, Santiago 7820436, Chile; 5 Electrical Engineering Department, Universidad de Santiago de Chile, Av. Ecuador 3519, Santiago 9170124, Chile; pablo.adasme@usach.cl; 6 Department of Computing and Industries, Universidad Católica del Maule, Talca 3466706, Chile; dzabala@ucm.cl; 7 Department of Electrical Engineering, Universidad de Chile, Santiago 8370451, Chile; pablo.palacios@ug.uchile.cl (P.P.J.); cazurdia@ing.uchile.cl (C.A.-M.)

**Keywords:** sound source localization, microphone arrays, time delay estimation, eigenvalue decomposition, generalized cross-correlation, direction of arrival estimation

## Abstract

Multiple simultaneous sound source localization (SSL) is one of the most important applications in the speech signal processing. The one-step algorithms with the advantage of low computational complexity (and low accuracy), and the two-step methods with high accuracy (and high computational complexity) are proposed for multiple SSL. In this article, a combination of one-step-based method based on the generalized eigenvalue decomposition (GEVD), and a two-step-based method based on the adaptive generalized cross-correlation (GCC) by using the phase transform/maximum likelihood (PHAT/ML) filters along with a novel T-shaped circular distributed microphone array (TCDMA) is proposed for 3D multiple simultaneous SSL. In addition, the low computational complexity advantage of the GCC algorithm is considered in combination with the high accuracy of the GEVD method by using the distributed microphone array to eliminate spatial aliasing and thus obtain more appropriate information. The proposed T-shaped circular distributed microphone array-based adaptive GEVD and GCC-PHAT/ML algorithms (TCDMA-AGGPM) is compared with hierarchical grid refinement (HiGRID), temporal extension of multiple response model of sparse Bayesian learning with spherical harmonic (SH) extension (SH-TMSBL), sound field morphological component analysis (SF-MCA), and time-frequency mixture weight Bayesian nonparametric acoustical holography beamforming (TF-MW-BNP-AHB) methods based on the mean absolute estimation error (MAEE) criteria in noisy and reverberant environments on simulated and real data. The superiority of the proposed method is presented by showing the high accuracy and low computational complexity for 3D multiple simultaneous SSL.

## 1. Introduction

In recent years, the analysis of smart meeting room activities has been an important area in the acoustic signal processing, where the sound source localization (SSL) is one of these applications. In some scenarios such as smart meeting rooms, the speech signal for one speaker is overlapped with other speakers, which raised the multiple sound source localization challenge based on the overlapped speech signal. Therefore, the researchers proposed some algorithms for multiple simultaneous SSL in noisy and reverberant environments for indoor scenarios [1]. The SSL algorithms usually use the microphone arrays for improving the locations’ estimations accuracy in acoustical environments. For example, the generalized cross-correlation (GCC) algorithm estimates the speakers’ directions by calculating the time difference of arrival (TDOA) between the microphone pairs [2]. The steered response power (SRP) [3] and SRP-phase transform (SRP-PHAT) [4] methods estimate the locations by evaluating a cost function based on the probability of the speakers’ presences on different three-dimensional points in the acoustical environment.

Currently, some methods have been proposed for simplifying the SSL systems based on the single-speaker methods [5]. These algorithms are based on a hypothesis, where the speech signals are separated in short-time Fourier transform (STFT) domain for multiple speakers’ scenarios, where each time-frequency (TF) bin with high probability contains the signal of a single speaker, which is named as windowed-disjoint orthogonality (W-DO) property [6]. This hypothesis is faced with many challenges, where the recorded signals by microphones contain the environmental reverberation. For solving this problem, some of the recent research works [7,8] are independent of speech signal for using the W-DO property. For example, Nadiri et al. in the first step proposed a correlation evaluation for determining the single-source content and then, considering a repetitive process for detecting the other sources in multi-speakers’ scenarios [9]. Similar to this method, the relative harmonic coefficients algorithm was proposed as a pre-processing method in recent years for detecting the single-speaker frames, which can be implemented for multi-speakers’ conditions within an iterative process [10]. On the contrary, the traditional subspace methods localize the speakers’ locations directly by using an overlapped speech signals [11,12]. The multiple signal classification (MUSIC) algorithm as a subspace method is popular due to the easy implementation and high efficiency [13]. In addition, some of the methods use the ad-hoc microphone arrays based on their advantage in comparison with other microphone arrays for SSL [14].

In recent decades, the array with high number of microphones (more than 30 microphones) for recording the speech signals are widely considered for SSL [15,16]. The high number of microphones prepare the possibility of using a set of orthogonal spatial functions for decomposing the measured voice pressure in spherical harmonic domain (SHC) [17]. The precision of the localization algorithms can affect the performance of other speech processing applications. Therefore, the SSL algorithms should be designed in a way for localizing the 3D positions of multiple simultaneous speakers in noisy and reverberant environments by eliminating the spatial aliasing.

In the last two decades, much research has been performed on SSL applications. Nikolaos et al. presented the perpendicular cross-spectra fusion (PCSF) method in 2017 as a new algorithm for direction of arrival (DOA) estimation [18]. This algorithm contains the subsystems for DOA estimating, which prepare the candidate DOAs for each time-frequency (TF) points by a parallel processing. Mert et al. presented an extension of SRP method in 2018 as steered response power density (SRPD) and single-adaptive search method, which is called hierarchical grid refinement (HiGRID) for decreasing the source candidate points in searching space [19]. Ning et al. in 2018 proposed a new framework for binaural source localization, which combines the model-based information of source spectral features with deep neural networks (DNN) [20]. Huawei and Wei proposed a robust sparse method in 2019 for multiple SSL in indoor scenarios with 3D spherical microphone arrays, which trains the temporal extension of multiple response model of sparse Bayesian learning with spherical harmonic (SH) extension (SH-TMSBL) [21]. Bing et al. presented a time-frequency spatial classification (TF-Wise) method in 2019 for localization and estimating the number of speakers by using of microphone arrays in undesirable conditions [22]. Luka et al. proposed a passive 3D SSL method in 2020, which localizes the speakers by geometric configuration of 3D microphone arrays [23]. Ning et al. in 2021 presented a sound field morphological component analysis (SF-MCA) method in combination with an enhanced alternative direction method of multipliers (ADMM) for accurate SSL [24]. The circular microphone arrays are widely considered in multi-speaker applications due to the flexibility in speech signal analysis, but the accuracy of the SSL algorithms is strongly dependent to the physical properties of the microphones, the level of the noise-reverberation, and the number of speakers. To address this problem, Kunkun et al. in 2021 presented an indoor multiple SSL algorithm based on an acoustical holography beamforming (AHB) and Bayesian nonparametric (BNP) methods [25]. They proposed a BNP algorithm based on infinite Gaussian mixture model (IGMM) for estimating the DOAs of independent sources without any pre-information of the number of speakers. To decrease the reverberation effect, they proposed a robust TF bins selection based on mixture weight (MW) method and implementing the algorithm on the selected frames. The MUSIC method is known as a traditional algorithm for estimating the DOAs of multiple speakers due to the easy implementation, but its accuracy decreases in noisy environments. Yonggang et al. in 2021 proposed a novel MUSIC algorithm based on the sound pressure measurement by using the high number of microphones in noisy environments [26].

The aim of this research article is proposing a 3D multiple simultaneous SSL system based on the novel T-shaped circular distributed microphone array (DMA) in combination with generalized eigenvalue decomposition (GEVD) and adaptive GCC-PHAT/maximum likelihood (ML) methods (TCDMA-AGGPM) for undesirable environments with low complexity. The proposed SSL method should be able to localize the multiple simultaneous speakers in noisy and reverberant scenarios with high accuracy and low computational complexity. A novel distributed arrangement is proposed for microphone arrays, where a limited number of microphones are considered in each time frame for decreasing the computational complexity. A circular microphone array (CMA) in the center of the room is considered in combination with GCC algorithm for estimating the speakers’ directions based on the robust proposed processing in front of the noise and reverberation. In addition, the full-band recurrent neural networks (F-CRNN) algorithm [27] is selected for estimating the number of speakers. Therefore, the GCC method is adaptively implemented in combination with PHAT filter for reverberant environments and ML filter for noisy conditions [28] on the recorded microphone arrays’ signals for estimating the central speakers’ DOAs (DOA_C_). Therefore, the two closest T-shaped microphone arrays on the walls are selected for each speaker based on the estimated DOA_C_. One of the T-shaped microphone arrays is considered in combination with GEVD algorithm for vertical DOA estimation and the other T-shaped array for horizontal DOA estimation. The uncertainty area for central array, vertical array, and horizontal array are estimated by calculating the standard deviation (SD) of obtained DOAs for all three microphone arrays (central, horizontal, and vertical) on different time frames. The intersection between these three areas creates an area in 3D space, where the 3D speakers’ locations are estimated by calculating the closest point in this area to all three DOAs. This process in repeated for all speakers to estimate the 3D speakers’ locations. The primary results of the proposed method were presented at the EUSIPCO 2021 conference [29], where it was implemented on simulated data and was compared with some simple works. In this article, in addition to its complete mathematical expansion, we considered adaptive GCC method by using the PHAT and ML filters. In addition, the proposed method is evaluated on real data for different range of signal-to-noise ratio (*SNR*) and reverberation time ($RT_{60}$). Also, the proposed TCDMA-AGGPM algorithm is compared with HiGRID [19], SH-TMSBL [21], SF-MCA [24], and TF-MW-BNP-AHB [25] methods, where the presented algorithm not only localizes the speakers more accurately, but also decreases the computational complexity in comparison with previous works on real and simulated data. The strategy for selecting these methods was based on the accuracy and computational complexity for multiple SSL, which are two important parameters in sound source localization methods.

Section 2 includes the microphone signal models and the proposed T-shaped circular distributed microphone array. Section 3 shows the proposed 3D multiple simultaneous SSL algorithm based on the combination of GCC-PHAT/ML method with central circular microphone array and GEVD algorithm with T-shaped microphone arrays. In Section 4, the results of the evaluations for the proposed TCDMA-AGGPM method are presented in comparison with HiGRID, SH-TMSBL, SF-MCA, and TF-MW-BNP-AHB algorithms on real and simulated data. Section 5 includes some conclusions of the presented algorithm for multiple SSL.

## 2. Distributed Microphone Array

The microphone arrays are frequently considered as an appropriate tool in the speech signal processing. Increasing the number of microphones in SSL algorithms covers a wider range of acoustical environments, where the localization methods estimate the speakers’ locations with equal accuracy for all speakers. In this section, the microphone signal models are presented for multiple simultaneous SSL applications. In addition, the proposed distributed microphone array is proposed based on the circular and T-shaped arrays.

### 2.1. Microphone Signal Model in SSL Applications

**Figure 1:**
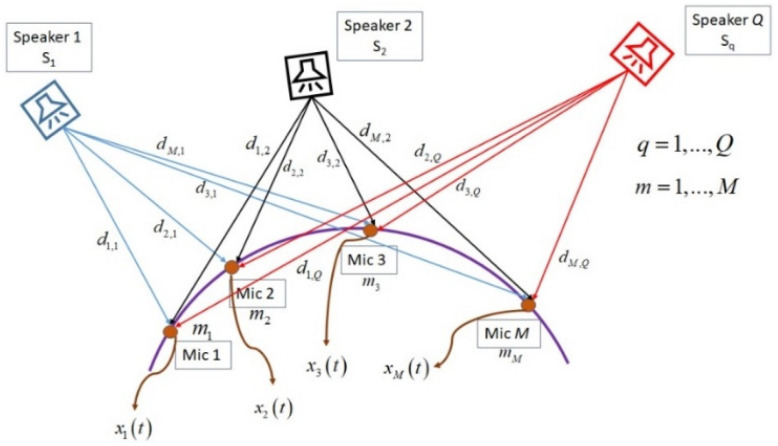
The relation between sound signals and microphones in near-field assumption for multiple speakers.

Microphone signal modelling is an important processing in the implementation of SSL algorithms on simulated data. The aim of this modeling is preparing the simulated data as much as possible similar to real recorded speech. Noise and reverberation are the undesirable environmental factors, where they effect the microphone signals and the accuracy of the speech processing algorithms. In acoustic applications, two microphone signal models are considered for SSL methods: 1-ideal model, and 2-real model. In an ideal model, the received signal by microphone is a delayed and weakened version of the speech source signal, which is expressed as:

(1) $$x_{m}^{I}\left(t\right)=\sum_{q=1}^{Q}x_{m,q}\left(t\right)=\sum_{q=1}^{Q}\frac{1}{d_{m,q}}s_{q}\left(t-\tau_{m,q}\right)+v_{m}\left(t\right)\;\left\{\forall m|m=1,\ldots ,M\right\},$$

where in Equation (1), $x_{m}^{I}\left(t\right)$ is the ideal received signal in the *m*-th microphone, $s_{q}\left(t\right)$ is the transmitted sound signal by *q*-th sound source, $\tau_{m,q}$ is the time delay between *q*-th sound source and *m*-th microphone, $d_{m,q}$ is the distance between *q*-th sound source and *m*-th microphone, $v_{m}\left(t\right)$ is the additive Gaussian noise in the *m*-th microphone, *M* is the number of microphones, and *Q* is the number of sound sources. Figure 1 shows the near-field model for the speech signal propagation from sound sources to the microphones.

This model is called ideal because the reverberation, which is an important undesirable factor, has not been considered in the formulations. The presented model for microphone signals should contain all undesirable factors to be similar to the real scenarios. Therefore, the real model is selected for the simulations of microphone signals. By considering the room impulse response (RIR), the real model is written as:

(2) $$x_{m}^{R}\left(t\right)=\sum_{q=1}^{Q}x_{m,q}\left(t\right)=\sum_{q=1}^{Q}s_{q}\left(t\right)*\gamma_{m,q}\left({\vec{d}}_{m,q},t\right)+v_{m}\left(t\right)\;\left\{\forall m|m=1,\ldots ,M\right\},$$

where in Equation (2), $x_{m}^{R}\left(t\right)$ is the real received signal in the *m*-th microphone, $\gamma_{m,q}\left({\vec{d}}_{m,q},t\right)$ is the RIR between *q*-th sound source and *m*-th microphone, and * denotes to convolution operator. By considering this model, the simulated signals are similar to real recorded speech signals in the environment, which is selected for the simulations in this article. In this model, the sound sources are independent, and noise is assumed as an additive signal in microphones’ places.

### 2.2. The Proposed T-Shaped Circular Distributed Microphone Array for SSL

**Figure 2:**
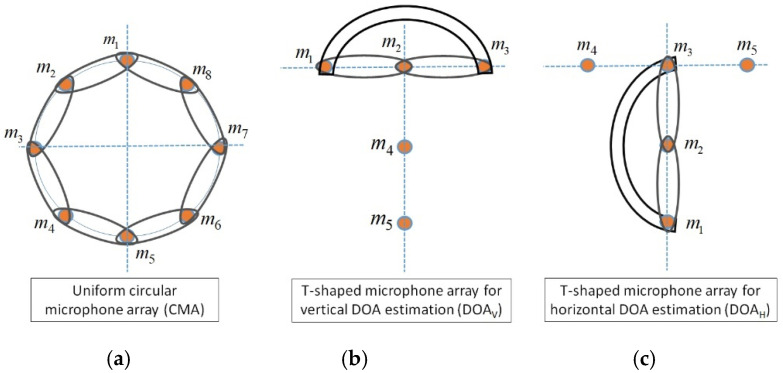
The proposed structure of T-shaped circular distributed microphone array for SSL, (**a**) central uniform circular array (in combination with GCC-PHAT/ML method), the T-shaped microphone array for (**b**) vertical DOA estimation (DOA_V_) (in combination with GEVD method), and (**c**) horizontal DOA estimation (DOA_H_) (in combination with GEVD method).

A microphone array uses a set of microphones, where they are located in some specific positions for recording an appropriate spatial information, which is called spatial diversity in wireless telecommunications. This diversity is represented by using the sound channel impulse response, which is the sound propagation path from sound source to microphone. These sound channels are modeled by finite impulse response (FIR) filters, which are not identical in general conditions. The microphone arrays prepare extra information, where the main issue in the microphone signal processing is estimating the parameters such as speakers’ locations or extracting some favorite signals in the speech enhancement applications. The microphone array geometry plays an important role in formulating the sound processing algorithms. For example, in SSL applications, the geometry of the microphone array must be known for estimating the correct speakers’ locations. In this article, a DMA is proposed as an appropriate solution for increasing the accuracy and decreasing the computational complexity of SSL algorithms. This proposed DMA is structured as a central uniform circular microphone array in combination with six T-shaped microphone arrays on the walls. Figure 2 shows the structure of circular and T-shaped microphone arrays. The circular microphone array in Figure 2a is selected in combination with adaptive GCC-PHAT/ML algorithm for estimating the central speakers’ directions (DOA_C_). Since the number of speakers are estimated by the F-CRNN [27] algorithm, the direction of each speaker is estimated by the proposed algorithm based on this circular array, which decreases the computational complexity. In the following, the T-shaped microphone arrays are selected in the second step in combination with GEVD algorithm, where the two closest T-shaped arrays to each speaker are selected as the input signals for GEVD algorithm. Each T-shaped microphone array is independently selected by the GEVD method, where the T-shaped microphone array in Figure 2b is considered for vertical DOA estimation (DOA_V_), and the T-shaped microphone array in Figure 2c for horizontal DOA estimation (DOA_H_). By considering an uncertainty area (β) around each estimated direction, three areas, $\beta_{C},\beta_{H},$ and $\beta_{V}$, are constructed around the estimated directions by these three microphone arrays. The intersection between these areas is considered for SSL, which is explained in the next section. The DMA prepares the condition for using the arrays in parallel and independently, where the central microphone array in combination with adaptive GCC-PHAT/ML algorithm is used simultaneously with each T-shaped microphone array in combination with GEVD algorithm, which decreases the implementation’s computational complexity. In addition, Figure 2 shows the selected microphone pairs for adaptive GCC-PHAT/ML and GEVD algorithm, which prepare the appropriate information for SSL process.

## 3. The Proposed SSL Algorithm in Combination with Distributed Microphone Array

**Figure 3:**
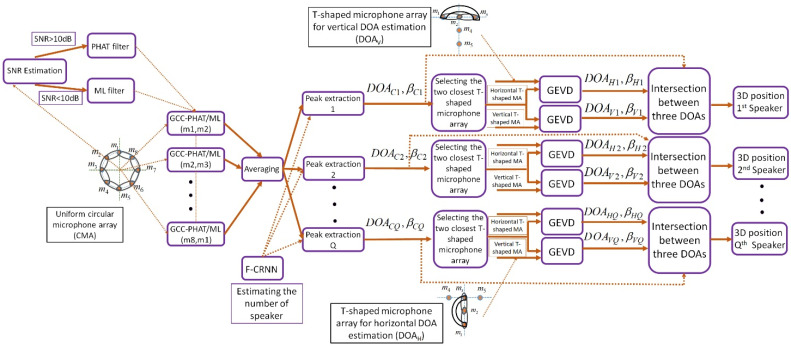
The block diagram of the proposed 3D multiple simultaneous SSL algorithm based on T-shaped circular distributed microphone array, adaptive GCC-PHAT/ML, and GEVD algorithms.

The multiple simultaneous SSL algorithms are divided into one-step and two-step methods. In two-step methods, the time delays are calculated between the microphone pairs and then, the speakers’ directions are estimated based on the microphone array geometry. This category of methods localizes the speakers with low computational complexity (faster) and low accuracy. The one-step methods are designed based on the propagated energy of each source. By considering a cost function, the candidate points in the environment are selected for maximizing or minimizing this cost function. These methods localize the speakers more accurately with high computational complexity (slower). In this article, a novel 3D multiple simultaneous SSL algorithm is proposed based on the TCDMA in combination with adaptive GCC-PHAT/ML and GEVD methods in noisy and reverberant environments. The proposed DMA provides an appropriate information in all room dimensions, which increases the accuracy and precision of SSL algorithm. In addition, the combination of adaptive GCC-PHAT/ML algorithm due to low complexity and GEVD method due to high accuracy is selected for proposing the novel SSL system. Figure 3 shows the block diagram of the proposed TCDMA-AGGPM algorithm, where each part of the system is explained in the following.

The first step of the proposed system is CMA, which is located in the room center. This CMA in combination with T-shaped arrays is called DMA, which are the main recording sections for preparing the signals for SSL processing. The microphone pairs in CMA provide the required signals for estimating the number of speakers in combination with adaptive GCC-PHAT/ML algorithm. In this article, the number of speakers is estimated by F-CRNN [27] algorithm based on the recorded signals by CMA. The GCC is an appropriate function for estimating the TDOAs between microphone pairs. The estimated TDOAs by this function are considered for estimating the speakers’ directions. As shown in Figure 1, $d_{m,q}$ is the distance between *q*-th sound source and *m*-th microphone. The relation between this distance and propagation delay for speech signal is formulated as:

(3) $$\tau_{m,q}=\frac{d_{m,q}}{C},$$

where in Equation (3), $\tau_{m,q}$ is the time delay between *q*-th sound source and *m*-th microphone, and *C* is the sound velocity. In addition, the related TDOAs for microphone pairs $\left\{m_{a},m_{b}\right\}$ and *q*-th sound source is called $\tau_{ab,q}$, which is simply expressed as the difference between propagation delays as:

(4) $$\tau_{ab,q}=\tau_{a,q}-\tau_{b,q}.$$

By replacing Equation (4) to Equation (3), the estimated TDOA for *q*-th sound source is formulated as the distance between sound source and microphone as:

(5) $$\tau_{ab,q}=\frac{d_{a,q}-d_{b,q}}{C},$$

where $d_{a,q}$ and $d_{b,q}$ are the distance between *q*-th source and microphones $m_{a}$ and $m_{b}$, respectively. Therefore, the source location is parametrized and estimated with some algorithms, where they consider these TDOAs for location estimation. If the real model is selected for simulations, the microphone signals $m_{a}$ and $m_{b}$ are expressed as [1]:

(6) $$x_{a}\left(t\right)=\sum_{q=1}^{Q}x_{ma,q}\left(t\right)=\sum_{q=1}^{Q}s_{q}\left(t\right)*\gamma_{ma,q}\left({\vec{d}}_{ma,q},t\right)+v_{ma}\left(t\right),$$

and,

(7) $$x_{b}\left(t\right)=\sum_{q=1}^{Q}x_{mb,q}\left(t\right)=\sum_{q=1}^{Q}s_{q}\left(t\right)*\gamma_{mb,q}\left({\vec{d}}_{mb,q},t\right)+v_{mb}\left(t\right).$$

The GCC function is the CC of filtered version of microphone signals $x_{a}\left(t\right)$ and $x_{b}\left(t\right).$ Based on the recorded signals by microphones $m_{a}$ and $m_{b}$, and by considering the Fourier transform for these filters as $G_{a}\left(\omega \right)$ and $G_{a}\left(\omega \right)$, the GCC function is expressed as:

(8) $$P_{ab}\left(\tau_{ab}\right)=\frac{1}{2\pi }\int_{-\infty }^{+\infty }\left(G_{a}\left(\omega \right)X_{a}\left(\omega \right)\right){\left(G_{b}\left(\omega \right)X_{b}\left(\omega \right)\right)}^{\prime }e^{j\omega \tau_{ab}}d\omega .$$

where $X_{a}\left(\omega \right)$ is the Fourier transform of signal $x_{a}\left(t\right)$ and $X_{b}\left(\omega \right)$ is the complex conjugate of Fourier transform of signal $x_{b}\left(t\right).$ By defining the weighting function $\psi_{ab}\left(\omega \right)=G_{a}\left(\omega \right){G^{\prime }}_{b}\left(\omega \right)$, the GCC function is written as:

(9) $$P_{ab}\left(\tau_{ab}\right)=\frac{1}{2\pi }\int_{-\infty }^{+\infty }\psi_{ab}\left(\omega \right)X_{a}\left(\omega \right){X^{\prime }}_{b}\left(\omega \right)e^{j\omega \tau_{ab}}d\omega .$$

In this article, the PHAT and ML weighting functions are considered in combination with GCC algorithm for SSL application. It has been shown in [28] that the GCC function in combination with PHAT filter increases the accuracy of estimated locations in reverberant scenarios with SNR>10 dB as:

(10) $$P_{ab}^{PHAT}\left(\tau_{ab}\right)=\frac{1}{2\pi }\int_{-\infty }^{+\infty }\frac{1}{\left|X_{a}\left(\omega \right){X^{\prime }}_{b}\left(\omega \right)\right|}X_{a}\left(\omega \right){X^{\prime }}_{b}\left(\omega \right)e^{j\omega \tau_{ab}}d\omega .$$

The GCC-PHAT function performs well in reverberant environments, but its accuracy decreases in noisy conditions. By experiments in [28], it has been shown that the ML filter is more robust in noisy environments with SNR<10 dB. When the reverberation is low and the noise and speech signals are uncorrelated, the ML weighting function is an unbiased estimator, which is expressed by power spectrum of source signal s(t) and noise signals $v_{a}\left(t\right)$ and $v_{b}\left(t\right)$ as:

(11) $$\psi_{ab}^{ML}\left(\omega \right)=\frac{\left|X_{a}\left(\omega \right)\right|\left|X_{b}\left(\omega \right)\right|}{{\left|V_{b}\left(\omega \right)\right|}^{2}{\left|X_{a}\left(\omega \right)\right|}^{2}+{\left|V_{a}\left(\omega \right)\right|}^{2}{\left|X_{b}\left(\omega \right)\right|}^{2}}.$$

It is assumed that the power spectrum density (PDF) for noise signals ${\left|V_{a}\left(\omega \right)\right|}^{2}$ and ${\left|V_{b}\left(\omega \right)\right|}^{2}$ are estimated from the silent part of the signal by using VAD. Therefore, the GCC-ML function is expressed as:

(12) $$P_{ab}^{ML}\left(\tau_{ab}\right)=\frac{1}{2\pi }\int_{-\infty }^{+\infty }\frac{\left|X_{a}\left(\omega \right)\right|\left|X_{b}\left(\omega \right)\right|}{{\left|V_{b}\left(\omega \right)\right|}^{2}{\left|X_{a}\left(\omega \right)\right|}^{2}+{\left|V_{a}\left(\omega \right)\right|}^{2}{\left|X_{b}\left(\omega \right)\right|}^{2}}X_{a}\left(\omega \right){X^{\prime }}_{b}\left(\omega \right)e^{j\omega \tau_{ab}}d\omega .$$

In this article, by measuring the *SNR* in microphone signals, the GCC-PHAT function is considered for SNR>10 dB (reverberant scenario), and the GCC-ML function for SNR<10 dB(noisy scenario), which is called adaptive GCC-PHAT/ML algorithm in the following. The adaptive GCC-PHAT/ML function’s peaks are the TDOAs related to the microphone pairs. For calculating the speakers’ directions, the TDOA values ($\tau_{ab}$) can be converted to DOA values ($\theta_{ab}$) as:

(13) $$\tau_{ab}=\frac{d}{C}\sin\left(\theta_{ab}\right)\;\to \;\theta_{ab}=\arcsin\left(\frac{\tau_{ab}.C}{d}\right).$$

The adaptive GCC-PHAT/ML function is averaged on all microphone pairs (M=8) for decreasing the effect of noise and reverberation as:

(14) $$P^{PHAT/ML}\left(\theta \right)=\frac{1}{M}\sum_{m=1}^{M}\frac{1}{2\pi }\int_{-\infty }^{+\infty }\psi_{m,m+1}\left(\omega \right)X_{m}\left(\omega \right){X^{\prime }}_{m+1}\left(\omega \right)e^{j\omega \frac{d}{C}\sin\theta }d\omega .$$

In Equation (14), microphone $m_{9}$ is equal as $m_{1}$, which is at the end of cycle. In the following, the adaptive GCC-PHAT/ML function’s peaks are extracted based on the number of speakers (*Q*), which is estimated by the F-CRNN algorithm.

(15) $$\begin{array}{l} {\hat{\theta }}_{C1}=\underset{0\leq \theta \leq 2\pi }{\arg\max}P^{PHAT/ML}\left(\theta \right)\;\to {\mathrm{DOA}}_{C1}\\ {\hat{\theta }}_{C2}=\underset{\begin{array}{l} 0\leq \theta \leq 2\pi \\ \theta \neq {\hat{\theta }}_{C1} \end{array}}{\arg\max}P^{PHAT/ML}\left(\theta \right)\;\to {\mathrm{DOA}}_{C2}\\ \;\;\;\;\;\;.\;\;\;\;\;\;\;\;\;\;\;\;\;\;\;\;\;\;\;\;\;\;\;\;\;\;\;\;.\;\;\;\;\;\;\;\;\;\;\;\;\;\;\;\;\;\;\;\;\;\;\;.\;\;\;\;\;\;\;\;\;\;\;\;\;\;\;\;\;\;\;\;\;\;,\\ \;\;\;\;\;\;.\;\;\;\;\;\;\;\;\;\;\;\;\;\;\;\;\;\;\;\;\;\;\;\;\;\;\;\;.\;\;\;\;\;\;\;\;\;\;\;\;\;\;\;\;\;\;\;\;\;\;\;.\\ {\hat{\theta }}_{CQ}=\underset{\begin{array}{l} 0\leq \theta \leq 2\pi \\ \theta \neq {\hat{\theta }}_{C1},\ldots ,{\hat{\theta }}_{CQ-1} \end{array}}{\arg\max}P^{PHAT/ML}\left(\theta \right)\to {\mathrm{DOA}}_{CQ} \end{array}$$

where ${\hat{\theta }}_{C1},{\hat{\theta }}_{C2},\ldots ,{\hat{\theta }}_{CQ}$ are the speakers’ directions based on the central uniform circular microphone array. An uncertainty area ($\beta_{Cq}$) is defined for each speaker, where the direction for speaker is considered around this area. This uncertainty area prepares the possibility for making a range in three-dimensional space, which provides the conditions for 3D SSL with intersection by other uncertainty areas from T-shaped microphone arrays. This uncertainty area is estimated by calculating the SD of estimated directions for each speaker based on the microphone pairs as:

(16) $$\beta_{Cq}=\sqrt{\frac{1}{M}\sum_{m=1}^{M}{\left({\hat{\theta }}_{Cq,m}-{\hat{\theta }}_{Cq}\right)}^{2}}\;for\;q=1,\ldots ,Q,$$

where in Equation (16), ${\hat{\theta }}_{Cq,m}$ is the estimated direction for *q*-th source by using the microphone pairs {m,m+1}, and $\beta_{Cq}$ is the uncertainty area for *q*-th speaker’s direction (${\mathrm{DOA}}_{Cq}$). Therefore, a specific area in 3D space is generated for each speaker. These uncertainty areas are calculated for all speakers ($\beta_{C1},\beta_{C2},\ldots ,\beta_{CQ}$) and the direction of each speaker is considered around this area (${\mathrm{DOA}}_{C1}\pm \beta_{C1},{\mathrm{DOA}}_{C2}\pm \beta_{C2},\ldots ,{\mathrm{DOA}}_{CQ}\pm \beta_{CQ}$).

In the following, two closest T-shaped microphone arrays are selected for each speaker, which is repeated for all speakers separately. One of these T-shaped microphone arrays is selected for calculating the horizontal direction estimation (DOA_H_) and horizontal uncertainty area ($\beta_{H}$), and the other T-shaped microphone array for vertical direction estimation (DOA_V_) and vertical uncertainty area ($\beta_{V}$). As shown in Figure 2, three microphone pairs are selected for vertical DOA estimating (Figure 2b) and another three microphone pairs for horizontal DOA estimating (Figure 2c). These T-shaped microphone arrays are considered for estimating the horizontal (DOA_H_) and vertical (DOA_V_) speakers’ directions in combination with GEVD algorithm. Therefore, the proposed TCDMA-AGGPM algorithm is defined based on the T-shaped microphone arrays as an input for GEVD algorithm. The acoustic room is assumed as a linear time-invariant (LTI) system, where the relation between the microphones’ signals and RIR is expressed as:

(17) $${\underline{x}}_{a}{}^{T}\left(n\right){\underline{g}}_{b}={\underline{x}}_{b}{}^{T}\left(n\right){\underline{g}}_{a},$$

where in Equation (17), the microphone signal ${\underline{x}}_{m}\left(n\right)$ is considered as:

(18) $${\underline{x}}_{m}\left(n\right)={\left[x_{m}\left(n\right),x_{m}\left(n-1\right),\ldots ,x_{m}\left(n-D+1\right)\right]}^{T},\;for\;m=1,2,3.$$

where ${\underline{x}}_{m}\left(n\right)$ is the sample’s vector signal for *m*-th microphone in T-shaped microphone array, *T* denotes to vector transpose, and *D* is the length of the signal (samples), which is equal to RIR length as:

(19) $${\underline{g}}_{m}={\left[g_{m,0},g_{m,1}\ldots \ldots g_{m,D-1}\right]}^{T}\;,\;m=1,2,3.$$

Since there is a fact that ${\underline{x}}_{m}\left(n\right)={\underline{g}}_{m}*s\left(n\right)$, then the covariance matrix for three microphone pairs is expressed as:

(20) $$B=\left(\begin{array}{ccc} B_{x_{1}x_{1}} & B_{x_{1}x_{2}} & B_{x_{1}x_{3}}\\ B_{x_{2}x_{1}} & B_{x_{2}x_{2}} & B_{x_{2}x_{3}}\\ B_{x_{3}x_{1}} & B_{x_{3}x_{2}} & B_{x_{3}x_{3}} \end{array}\right),$$

where the covariance matrix elements are defined as $B_{x_{a}x_{b}}=E\left\{{\underline{x}}_{a}\left(n\right){\underline{x}}^{T}{}_{b}\left(n\right)\right\}\;,(a,b=1,2,3)$. In addition, vector *u* with length 3×D, which contains the impulse response for these three microphone pairs, is shown as:

(21) $$\underline{u}=[\begin{array}{c} {\underline{g}}_{3}\\ -{\underline{g}}_{2}\\ -{\underline{g}}_{1} \end{array}].$$

Vector *u* is the eigenvector of matrix *B* related to eigenvalue 0. In addition, if the impulse responses ${\underline{g}}_{1},{\underline{g}}_{2},$ and ${\underline{g}}_{3}$ do not have a common zero, and the covariance matrix of signal s(n) has complete order, the covariance matrix *B* has only one eigenvalue equal to 0. The exact estimation of vector *u* is impossible because of characteristics of speech signal, room impulse response length, background noise, etc. The robust GEVD method extracts the random gradient algorithms and estimates the generalized eigenvector related to the smallest generalized eigenvalue of noise covariance matrix ($B_{D}^{b}$) and signal covariance matrix ($B_{D}^{x}$), in an iterative process. It is assumed that the noise covariance matrix ($B_{D}^{b}$) is known, which is estimated from silence parts of the recorded signal. In addition, we assume that the noise is sufficiently stationary, where the noise covariance matrix, which is estimated from silence part of the signal, can be used for updating the formulas in the frames with mixture of the signal and noise. Instead of updating all GEVD functions for $B_{D}^{b}$, $B_{D}^{x}$ and estimating the generalized eigenvector related to smallest generalized eigenvalue, the generalized eigenvector is estimated by minimizing the cost function ${\underline{u}}^{T}B_{D}^{x}\underline{u}$ in an iterative process [30]. This low complexity method for minimizing the mean square error (MSE) of error signal e(n) is called Rayleigh Quotient, which is shown as:

(22) $$e(n)=\frac{{\underline{u}}^{T}\left(n\right){\underline{x}}_{D}\left(n\right)}{\sqrt{{\underline{u}}^{T}\left(n\right)B_{D}^{b}\underline{u}\left(n\right)}}=\frac{{\underline{u}}^{T}\left(n\right){\underline{x}}_{D}\left(n\right)}{\left||\sqrt{B_{D}^{b}\underline{u}\left(n\right)}|\right|}$$

Based on least mean square (LMS) adaptive filter, vector *u* is expressed as:

(23) $$\underline{u}\left(n+1\right)=\underline{u}\left(n\right)-\mu e\left(n\right)\frac{\partial \underline{u}\left(n\right)}{\partial e\left(n\right)},$$

where μ is adaptation step in LMS algorithm and the gradient of vector *u* is written as:

(24) $$\frac{\partial e\left(n\right)}{\partial \underline{u}\left(n\right)}=\frac{1}{\sqrt{{\underline{u}}^{T}\left(n\right)B_{D}^{b}\underline{u}\left(n\right)}}\left({\underline{x}}_{D}(n)-e\left(n\right)\frac{B_{D}^{x}\underline{u}\left(n\right)}{\sqrt{{\underline{u}}^{T}\left(n\right)B_{D}^{b}\underline{u}\left(n\right)}}\right).$$

By replacing Equations (22) and (24) in Equation (23), the vector *u* is expressed as:

(25) $$\underline{u}\left(n+1\right)=\underline{u}\left(n\right)-\frac{\mu }{{\underline{u}}^{T}\left(n\right)B_{D}^{b}\underline{u}\left(n\right)}\left[{\underline{x}}_{D}\left(n\right){\underline{x}}_{D}^{T}\left(n\right)\underline{u}\left(n\right)-e^{2}\left(n\right)B_{D}^{b}\underline{u}\left(n\right)\right].$$

By calculating the expected value (*E*) of covariance matrix, the vector *u* is written as:

(26) $$B_{D}^{x}\underline{u}\left(\infty \right)=E\left\{e^{2}\left(n\right)\right\}B_{D}^{b}\underline{u}\left(\infty \right),$$

where $\underline{u}\left(\infty \right)$ is the generalized eigenvector related to smallest generalized eigenvalue of covariance matrixes $B_{D}^{x}$ and $B_{D}^{b}$. To avoid the error in estimations, an extra normalization step is implemented in each repetition. Therefore, the impulse response vector *u* is formulated as:

(27) $$\tilde{\underline{u}}\left(n+1\right)=\underline{u}\left(n\right)-\mu e\left(n\right)\left\{{\underline{x}}_{D}\left(n\right)-e\left(n\right)B_{D}^{b}\underline{u}\left(n\right)\right\}.$$

Finally,

(28) $$\underline{u}\left(n+1\right)=\frac{\tilde{\underline{u}}\left(n+1\right)}{\sqrt{{\tilde{\underline{u}}}^{T}\left(n+1\right)B_{D}^{b}\tilde{\underline{u}}\left(n+1\right)}},$$

where vector *u* contains the impulse responses between source and selected microphones in T-shaped microphone array. By estimating the impulse responses ${\underline{g}}_{1},{\underline{g}}_{2},{\underline{g}}_{3}$, the horizontal (DOA_H_) and vertical (DOA_V_) speaker’s directions are calculated for a specific speaker. Based on the T-shaped microphone array in Figure 2b, which is considered for vertical direction estimating, the DOA_V_ is expressed as:

(29) $${\hat{\theta }}_{V,q}=\frac{1}{3}\sum_{k=1}^{3}\underset{V}{{\hat{\theta }}_{ab,k}}\;for\;\left\{ \begin{array}{l} a=1,\ldots ,3\\ b=1,\ldots ,3\\ q=1,\ldots ,Q \end{array}\right.,$$

and the uncertainty area ($\beta_{V}$) for vertical DOA estimation and *q*-th speaker is expressed as:

(30) $$\beta_{V,q}=\sqrt{\frac{1}{3}\sum_{k=1}^{3}{\left(\underset{V}{{\hat{\theta }}_{ab,k}}-{\hat{\theta }}_{V,q}\right)}^{2}}\;for\;\left\{ \begin{array}{l} a=1,\ldots ,3\\ b=1,\ldots ,3\\ q=1,\ldots ,Q \end{array}\right.,$$

This process is repeated for T-shaped microphone array in Figure 2c for calculating the horizontal speaker’s direction (DOA_H_) for *q*-th speaker as:

(31) $${\hat{\theta }}_{H,q}=\frac{1}{3}\sum_{k=1}^{3}\underset{H}{{\hat{\theta }}_{ab,k}}\;for\;\left\{ \begin{array}{l} a=1,\ldots ,3\\ b=1,\ldots ,3\\ q=1,\ldots ,Q \end{array}\right.,$$

Similarly, the uncertainty area ($\beta_{H}$) for horizontal direction estimations (DOA_H_) for *q*-th speaker is expressed as:

(32) $$\beta_{H,q}=\sqrt{\frac{1}{3}\sum_{k=1}^{3}{\left(\underset{H}{{\hat{\theta }}_{ab,k}}-{\hat{\theta }}_{H,q}\right)}^{2}}\;for\;\left\{ \begin{array}{l} a=1,\ldots ,3\\ b=1,\ldots ,3\\ q=1,\ldots ,Q \end{array}\right.,$$

Finally by calculating the speaker direction and its uncertainty area with central circular microphone array (DOA_C_ $\pm \;\beta_{C}$), for T-shaped microphone array in Figure 2b (DOA_V_ $\pm \;\beta_{V}$) and T-shaped microphone array in Figure 2c ( DOA_H_ $\pm \;\beta_{H}$) for *q*-th speaker, three areas are generated in three-dimensional space, where the 3D speakers’ locations are estimated by intersection between these three areas and calculating the closest point in the intersected area to all of them. This process is repeated for all *Q* speakers for calculating the exact 3D locations. The accurate and fast location estimation are provided in our proposed TCDMA-AGGPM method by considering the novel T-shaped circular distributed microphone array in combination with adaptive GCC-PHAT/ML and GEVD algorithms.

## 4. Results and Discussions

### 4.1. Data Recording and Simulation Conditions

**Figure 4:**
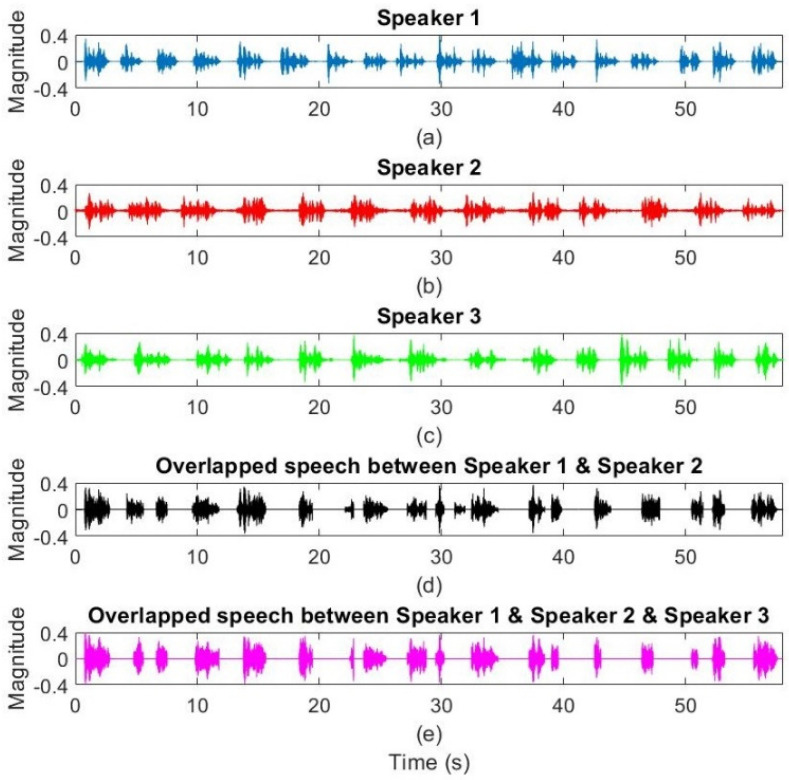
The time-domain speech signal for (**a**) 1st speaker (S1), (**b**) 2nd speaker (S2), (**c**) 3rd speaker (S3), (**d**) overlap between speakers S1 and S2, and (**e**) overlap between speakers S1, S2, and S3.

The proposed TCDMA-AGGPM method is evaluated on real and simulated data for covering all undesirable environmental scenarios. The Texas Instruments and Massachusetts Institute of Technology (TIMIT) dataset [31] is selected as an advanced bank of the speech signals for simulations. One female and two male speakers are selected for evaluating the proposed algorithm, where one male (S1) and one female (S2) speaker are considered for two simultaneous speakers’ scenarios, and all three speakers (S1, S2, and S3) are considered for the scenario with three speakers. In addition, the proposed algorithm is implemented on real recorded voice data at speech, music, and image processing laboratory (SMIPL), Universidad Tecnológica Metropolitana (UTEM), Santiago, Chile. The conditions for real data recording are the same as the simulated data. For example, two speakers were speaking simultaneously for two overlapped speakers’ scenario. In addition, all speakers are oriented to the central microphone array. Therefore, the results of evaluation can be extended to different conditions. The aim of the proposed method is 3D multiple simultaneous SSL for noisy and reverberant conditions in real scenarios. Various experiments have been performed on scenarios in smart meeting rooms. It has been shown in [32], where in real scenarios for conference events, around 90% of the overlapped signal are for two simultaneous speakers, 8% of the time for three overlapped simultaneous speakers, and the rest for four speakers and up. Therefore, the evaluations are structured for two and three simultaneous speakers for covering a wide range of meeting events in real environments. In the simulations, 58.84 seconds of speech signal are recorded for each speaker (S1, S2, and S3), where there are the silent areas in recorded signal, which are used for updating noise covariance matrix $B_{D}^{b}$ in the proposed algorithm. In addition, 26.80 and 21.57 seconds of the recorded signals belong to two (S1 and S2) and three (S1, S2, and S3) simultaneous speakers, respectively. Figure 4 shows the speech signals in time-domain for all three speakers, overlapped between two speakers (S1, and S2), and overlapped between three speakers (S1, S2, and S3). As shown in this figure, the percentage of overlapped signal between three speakers is less than the overlap between two speakers.

**Figure 5:**
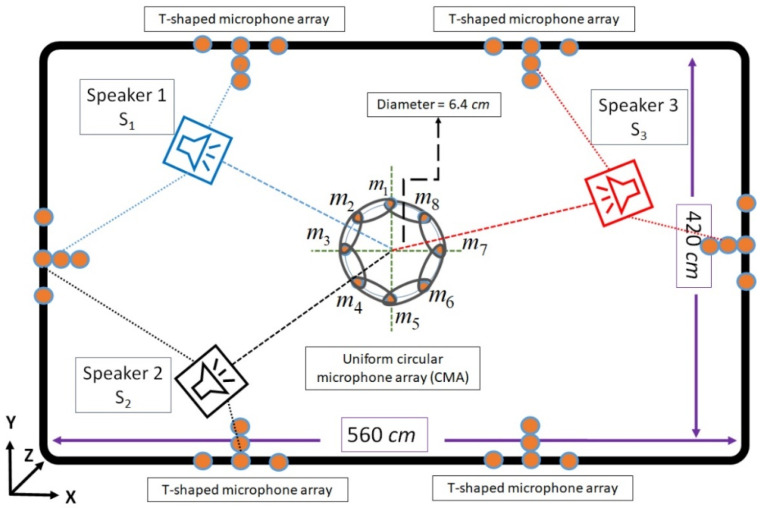
A view of the simulated room with speakers, circular, and T-shaped microphone arrays.

In addition, three speakers are located in the fixed positions in the acoustical room. The first, second, and third speakers are located at S1 = (115,327,183) cm, S2 = (13,684,165) cm, and S3 = (461,245,174) cm, respectively. The speakers’ locations are selected in a way for evaluating the proposed SSL algorithm at different angles in the room. The proposed DMA, which is the combination of eight microphones circular and T-shaped arrays, is an important step for preparing the proper signals for the proposed TCDMA-AGGPM algorithm. The inter-microphone distances are adjusted as *d* = 2.4 cm for avoiding the spatial aliasing between microphone signals in the proposed algorithm. In addition, six T-shaped microphone arrays with five microphones in each one is installed on the walls. Since the T-shaped microphone arrays play the main role in 3D SSL algorithm, the best places on the walls are considered for the installation and covering all room angles. Figure 5 shows a view of the simulated room with the speakers’ locations and microphones. In addition, the exact location of microphones and speakers with room dimensions are reported in Table 1.

### 4.2. The Evaluation’s Scenarios

The environmental undesirable factors decrease the accuracy and precision of the SSL algorithms in real scenarios. Noise, reverberation, and spatial aliasing are the most important undesirable factors in speech recording scenarios. The spatial aliasing is eliminated with proper placement of microphones by inter-microphone distance calculation based on the Nyquist theorem. In addition, the proposed TCDMA avoids the spatial aliasing because the accurate localization is provided by placing the microphones close to each other and considering the near-field assumption. On the contrary, noise and reverberation are the permanent undesirable factors in acoustical environments, which is impossible to eliminate completely. The white Gaussian noise (WGN) is adaptively considered in the microphones’ places for the simulations. The WGN is similar to real noise in acoustical environments and the recorded signals in SMIPL at UTEM. The Image model [33] is selected for simulating the reverberation effects in the evaluations. This model provides an estimation of RIR similar to real scenarios. This model generates the impulse responses between sound source and microphone by considering the microphone place, source location, room dimensions, impulse response length, sampling frequency, environmental reflection coefficients, and reverberation time ($RT_{60}$). The recorded microphone’s signal is generated by convolution between source signal and produced RIR by Image method. This process is repeated for all microphones and sources to generate the simulated signals. In addition, the Hamming window with 60 ms length [34] is selected for providing the stationary samples of speech signal in each time frame, which is an optimal length in SSL applications. Also, 50% overlap between time frames is considered for taking advantage of the most appropriate recorded speech signals parts. The sampling frequency is considered as $F_{s}=16000\;\mathrm{Hz}$, which is popular in speech processing applications for teleconferencing. In simulations, the length of room impulse response is selected as D=960 samples, where the length of *u* vector is 2880 samples. Also, the adaptation step in GEVD algorithm is assumed as $\mu =10^{-7}$, which provides the fast and appropriate convergence for adaptive filters. The simulations are performed by MATLAB software, version 2021b (MathWorks, Natick, MA, USA). In addition, the algorithms are implemented on a laptop with CPU core i7-10875H (Intel, Santa Clara, CA, USA), 2.3 GHz, and 64 GB RAM. The proposed TCDMA-AGGPM algorithm is compared with HiGRID [19], SH-TMSBL [21], SF-MCA [24], and TF-MW-BNP-AHB [25] methods for two and three simultaneous speakers in noisy and reverberant environments on real and simulated data. The mean absolute estimation error (MAEE) [35] criteria is selected for measuring the accuracy and robustness of the proposed TCDMA-AGGPM method in comparison with other previous works. This criteria provides a measurement scale by calculating the accurate distance between 3D estimated speaker’s location (${\hat{x}}_{q},{\hat{y}}_{q},{\hat{z}}_{q}$) and real speaker’s location ($x_{q},y_{q},z_{q}$) with averaging on $N_{t}$ continuous frames of overlapped speech signal, which is expressed as:

(33) $${\mathrm{MAEE}}_{q}=\frac{1}{N_{t}}\sum_{i=1}^{N_{t}}\left|\left(x_{q,i},y_{q,i},z_{q,i}\right)-\left({\hat{x}}_{q,i},{\hat{y}}_{q,i},{\hat{z}}_{q,i}\right)\right|,$$

where in Equation (33), ($x_{q,i},y_{q,i},z_{q,i}$) is the *q*-th real speaker’s location, and (${\hat{x}}_{q,i},{\hat{y}}_{q,i},{\hat{z}}_{q,i}$) is the *q*-th estimated speaker’s location in *i*-th time frames.

### 4.3. The Results on Simulated and Real Data

The simulations are designed for two and three simultaneous speakers on noisy and reverberant environments to cover a wide range of real scenarios. Therefore, two categories of evaluations are considered for comparison between the proposed TCDMA-AGGPM and other previous works. In the first category, the proposed method is implemented on a series of defined real environmental scenarios, which happen frequently in real conditions. In the second category of evaluations, the precision and accuracy of the proposed method in the first step, is evaluated for fixed *SNR* and variable $RT_{60}$, and in the second step on fixed $RT_{60}$ and variable *SNR*. For the first category, three environmental scenarios are defined for the evaluations. The first scenario is called reverberant environment by SNR=20 dB and $RT_{60}=650\;\mathrm{ms}$. The second scenario is noisy environment, where the effect of the noise is dominant by SNR=5 dB and $RT_{60}=250\;\mathrm{ms}$. The third scenario is named noisy-reverberant environment by SNR=5 dB and $RT_{60}=650\;\mathrm{ms}$, which is very challenging for most of the SSL algorithms.

Table 2 shows the MAEE results in cm for the proposed TCDMA-AGGPM algorithm in comparison with HiGRID, SH-TMSBL, SF-MCA, and TF-MW-BNP-AHB methods for two simultaneous speakers, on real and simulated data for reverberant, noisy, and noisy-reverberant scenarios. In each part of this table, the results are reported separately for each speaker (S1 and S2) to show the accuracy and robustness of the proposed method. As shown in this table, the HiGRID algorithm localizes the speakers less accurate in comparison other works. After that, the SH-TMSBL and SF-MCA algorithms prepared the better results for SSL. The proposed TCDMA-AGGPM algorithm is in competition with TF-MW-BNP-AHB method, where our proposed method localizes the speakers more accurate, but in some scenarios the results of these two methods are very similar. For example, in reverberant environment (scenario 1) and for simulated data, the MAEE criteria for proposed TCDMA-AGGPM and TF-MW-BNP-AHB methods are 32 and 36 cm for speaker S1, respectively, and the same results are 35 and 38 cm for speaker S2. In addition, in reverberant scenario and real data, the MAEE criteria for proposed TCDMA-AGGPM and TF-MW-BNP-AHB methods are 34 and 39 cm for speaker S1, and 37 and 41 cm for speaker S2, respectively. In addition, in noisy-reverberant environment and for simulated data, the MAEE criteria for proposed TCDMA-AGGPM and TF-MW-BNP-AHB methods are 42, and 47 cm for speaker S1, respectively, and the same results are 45 and 52 cm for speaker S2. In noisy-reverberant scenario and real data, the MAEE criteria for proposed TCDMA-AGGPM and TF-MW-BNP-AHB methods are 44 and 55 cm for speaker S1, and 47 and 58 cm for speaker S2, respectively Also, the other results in this table show the superiority of the proposed method for two simultaneous speakers in comparison with other previous works on real and simulated data for reverberant, noisy, and noisy-reverberant scenarios.

**Figure 6:**
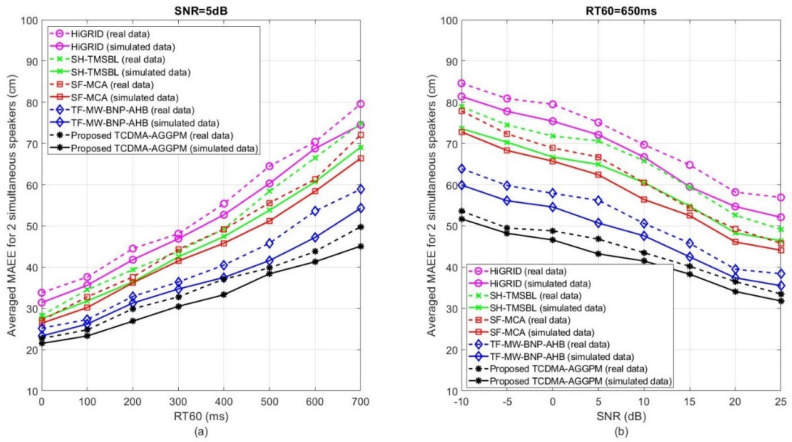
The averaged MAEE results (in cm) for the proposed TCDMA-AGGPM algorithm in comparison with HiGRID, SH-TMSBL, SF-MCA, and TF-MW-BNP-AHB methods, for 2 simultaneous speakers on real and simulated data, (**a**) for SNR=5 dB and $0\leq RT_{60}\leq 700\;\mathrm{ms}$, and (**b**) for $RT_{60}=650\;\mathrm{ms}$ and −10 dB ≤ *SNR* ≤ 25 dB.

The second category of comparisons are the accuracy and precision measurements based on the variation of noise and reverberation. Therefore, these scenarios are designed in a way for evaluating first, for fixed *SNR* and variable $RT_{60}$, and second, for the fixed $RT_{60}$ and variable *SNR*. In addition, the MAEE criteria is implemented by averaging on 25 time frames for preparing the reliable results. Figure 6 shows the averaged MAEE results for the proposed TCDMA-AGGPM algorithm in comparison with HiGRID, SH-TMSBL, SF-MCA, and TF-MW-BNP-AHB methods for two simultaneous speakers on real and simulated data. Figure 6a represents the results for SNR=5 dB and $0\leq RT_{60}\leq 700\;\mathrm{ms}$ on real (dash line) and simulated (solid line) signals. As shown in this figure, the HiGRID and our proposed TCDMA-AGGPM methods obtain the highest (lowest accuracy) and lowest (highest accuracy) MAEE values in comparison with other methods, respectively. This figure shows that the accuracy of all methods decreases by increasing the $RT_{60}$ value. In addition, almost in all methods, the real data has lesser accuracy in comparison with simulated data, because controlling the undesirable factors are easier in simulated conditions in comparison with real scenarios. In some cases, even measuring the *SNR* and $RT_{60}$ for real data is a challenge in the evaluations, which is performed with some error. The results of our proposed TCDMA-AGGPM algorithm are closer to the TF-MW-BNP-AHB method, where in $RT_{60}=100\mathrm{ms}$, the averaged MAEE value for our proposed algorithm and TF-MW-BNP-AHB method are 23 and 26 cm, and in $RT_{60}=600\;\mathrm{ms}$ are 41 and 47 cm for simulated data, respectively, where in both cases our proposed method localizes the speakers with higher accuracy in comparison with other previous works. Figure 6b similarly shows the results for $RT_{60}=650\;\mathrm{ms}$ and −10 dB≤SNR≤25 dB for two simultaneous speakers on real and simulated data. As shown in this figure, the accuracy of SH-TMSBL and SF-MCA methods are similar, but the proposed TCDMA-AGGPM algorithm localizes the speakers more accurately in comparison with other previous works. For example, the averaged MAEE criteria for simulated data in SNR=5 dB for the proposed TCDMA-AGGPM is 43 cm, the TF-MW-BNP-AHB method is 50 cm, and for HiGRID, SH-TMSBL, and SF-MCA algorithms are 72, 64, and 62 cm, respectively. These values show the superiority of the proposed method in comparison with other previous works for variable $RT_{60}$ in two speakers’ scenarios. As presented in this figure, all methods contain better accuracy in higher *SNR*s and weaker accuracy in lower *SNR*s. This means noise highly decreases the accuracy of the localization algorithm. It is important to consider that SNR=5 dB and $RT_{60}=650\;\mathrm{ms}$ at the same time generates a very undesirable noisy and reverberant scenario, which rarely happens in some specific cases in the real environments.

Table 3 shows similar results of MAEE criteria for the proposed TCDMA-AGGPM algorithm in comparison with HiGRID, SH-TMSBL, SF-MCA, and TF-MW-BNP-AHB methods for three simultaneous speakers on real and simulated data for reverberant (scenario 1), noisy (scenario 2), and noisy-reverberant (scenario 3) environments. As shown in this table, the proposed method localizes the speakers more accurately in comparison with other previous works. The accuracy of the methods is higher in noisy scenario, decreases for reverberant and noisy-reverberant conditions, which are the conditions with the lowest accuracy and precision. For example, on simulated data for noisy-reverberant scenario and for the third speaker (S3), the proposed method localizes the speaker with MAEE equal to 46 cm in comparison with HiGRID by 77 cm, SH-TMSBL by 70 cm, SF-MCA by 65 cm, and TF-MW-BNP-AHB method by 54 cm, which clearly shows that the proposed TCDMA-AGGPM algorithm localizes the speakers more accurately in comparison with other previous works, especially in noisy-reverberant environments. The second part in this table is related to real data, which contain the lower accuracy in comparison with simulated data based on the mentioned reason. In addition, the proposed method localizes the speakers more accurately even in real data. For example, in the third scenario for the third speaker, the MAEE value for proposed TCDMA-AGGPM, HiGRID, SH-TMSBL, SF-MCA, and TF-MW-BNP-AHB methods are 48, 78, 73, 70, and 59 cm respectively, which clearly shows the superiority of the proposed method in comparison with other previous works.

**Figure 7:**
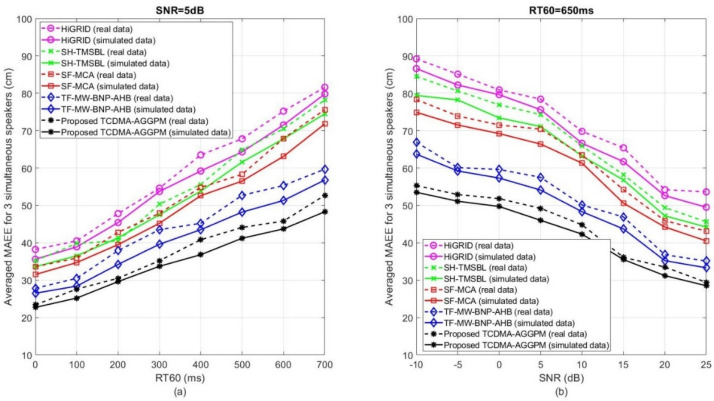
The averaged MAEE results (in cm) for the proposed TCDMA-AGGPM algorithm in comparison with HiGRID, SH-TMSBL, SF-MCA, and TF-MW-BNP-AHB methods, for 3 simultaneous speakers on real and simulated data, (**a**) for SNR=5 dB and $0\leq RT_{60}\leq 700\;\mathrm{ms}$, and (**b**) for $RT_{60}=650\;\mathrm{ms}$ and −10 dB≤SNR≤25 dB.

Figure 7 shows the averaged MAEE values for the proposed TCDMA-AGGPM algorithm in comparison with HiGRID, SH-TMSBL, SF-MCA, and TF-MW-BNP-AHB methods for three simultaneous speakers on real and simulated data for different ranges of *SNR* and $RT_{60}$ to evaluate the precision and robustness of the algorithms in noisy and reverberant scenarios. Figure 7a shows the results for SNR=5 dB and $0\leq RT_{60}\leq 700\;\mathrm{ms}$ on real (dash line) and simulated (solid line) data. As shown in this figure, the proposed TCDMA-AGGPM algorithm has lower averaged MAEE values in comparison with other previous works, which means that the algorithm localizes the speakers more accurately. For example, in $RT_{60}=100\;\mathrm{ms}$, the proposed TCDMA-AGGPM method localizes the speaker with averaged MAEE equal to 25 cm, where its accuracy is higher in comparison with the best other previous works like TF-MW-BNP-AHB method with 29 cm error on simulated data. In addition, the averaged MAEE in $RT_{60}=600\;\mathrm{ms}$ for proposed TCDMA-AGGPM and TF-MW-BNP-AHB methods are 44 and 51 cm, respectively, which shows the superiority of our proposed method in high reverberant scenario. Also, this figure represents that the accuracy of all methods decreases by increasing the reverberation time and the real data has lower accuracy in comparison with simulated data. Figure 7b shows the averaged MAEE values for $RT_{60}=650\;\mathrm{ms}$ and −10 dB≤SNR≤25 dB in three speakers’ scenario. As represented in this figure, the proposed TCDMA-AGGPM method localizes the speakers more accurately in comparison with HiGRID, SH-TMSBL, SF-MCA, and TF-MW-BNP-AHB algorithms. For example, in SNR=5 dB,the averaged MAEE value for the proposed method is 46 cm in comparison with TF-MW-BNP-AHB algorithm with 54 cm, where the other algorithms localize speakers less accurately. Most of the methods have higher accuracy in high *SNR*s, but the proposed method with averaged MAEE 31 cm even works better in comparison with TF-MW-BNP-AHB algorithm with 35 cm in SNR=20 dB. In addition, this figure clearly shows that the accuracy of all methods decreases in low *SNR*s, and the simulated data has better results in comparison with real data. These results show the superiority of the proposed TCDMA-AGGPM algorithm in comparison with other previous works. Our localization method can have a challenge if two speakers are exactly in the same direction to the central microphone array with different distances. In this condition, the algorithm may estimate the position of one the speakers wrongly. This scenario happens just in the case the two speakers are speaking at the same time and they are in the same direction. For this reason, we avoid the speakers to be in the same direction at the same time.

Computational complexity is an important parameter for implementing the SSL algorithms in real scenarios. The algorithms with high level of complexity are not able to practically localize the speakers in real conditions. Most of the SSL algorithms only increase the accuracy of estimated locations without attending to the complexity, which makes the method unimplementable in real scenarios. In this article, the MATLAB run-time in seconds is considered as a scale for comparing the complexity of the algorithms. Table 4 shows the program’s run-time in seconds for the proposed TCDMA-AGGPM algorithm in comparison with HiGRID, SH-TMSBL, SF-MCA, and TF-MW-BNP-AHB methods for two and three simultaneous speakers in noisy-reverberant environments on real data. As shown in this table, the HiGRID and SH-TMSBL methods require more time for localizing the speakers, which means more calculating in programming, but the SF-MCA and TF-MW-BNP-AHB algorithms localize the speakers with less complexity. The proposed TCDMA-AGGPM algorithm decreases the computational complexity due to parallel signal processing in combination with using the uniform CMA as a part of DMA and a T-shaped microphones on the walls, where both arrays are performing separately at the same time. This important advantage prepares the condition for implementing the proposed algorithm in real environments, which is critical in pseudo real-time systems. The program’s run-time can be decreased by using faster processors, which is an important improvement for future works. Based on the results in the last figures and tables, not only does the proposed TCDMA-AGGPM method localize the simultaneous speakers in three-dimensions with more accuracy in noisy and reverberant scenarios, but it also highly decreases the computational complexity of 3D SSL, which is an important advantage in implementing the 3D simultaneous SSL algorithms in real scenarios.

## 5. Conclusions

The 3D multiple simultaneous SSL is one of the most important and challenging topics in the speech processing applications. The accuracy and precision of most algorithms are decreased in noisy and reverberant conditions. In this article, a novel 3D multiple simultaneous SSL algorithm was proposed based on the T-shaped circular DMA in combination with GEVD and adaptive GCC-PHAT/ML methods for noisy and reverberant environments. The proposed TCDMA array provided more accurate locations’ estimations with low computational complexity. Firstly, the central uniform CMA is considered in combination with GCC method for estimating the speakers’ directions. In addition, the weighing PHAT and ML filters are adaptively implemented based on the *SNR* of recorded signals for decreasing the undesirable environmental factors. Then, the two closest T-shaped arrays are selected for each speaker due to the directions’ estimations in the first step. Each of these two T-shaped arrays is considered in combination with GEVD algorithm for estimating the horizontal and vertical directions, respectively. An uncertainty area (β) is selected based on the SDs of estimated directions of microphone pairs for circular ($\beta_{C}$), horizontal ($\beta_{H}$), and vertical ($\beta_{V}$) T-shaped microphone arrays around the estimated DOAs. Finally, the 3D location of each speaker is estimated by intersection between these three areas and finding the closest point to all DOAs. The proposed TCDMA-AGGPM algorithm was compared with HiGRID, SH-TMSBL, SF-MCA, and TF-MW-BNP-AHB methods based on the averaged MAEE criteria for two and three simultaneous speakers. In addition, the proposed method localizes the speakers with less complexity in comparison with other previous works based on the measured program’s run-time. The only disadvantage of this method is the primary installation cost, since we use 38 microphones in both T-shaped and circular microphone arrays, which is higher in comparison with other previous works.

One of the important fields for the future work in this research area is reviewing the other microphone arrays in combination with sound source localization algorithms. Decreasing the number of microphones without affecting the localization accuracy is considered as an aim of the future work in this SSL application because it can decrease the installation cost. In addition, increasing the accuracy of this SSL algorithm by using some subband techniques in noisy and reverberant environment is another area for future work. 

## Figures and Tables

**Table 1:** The exact locations of speakers, circular microphones, and room dimensions.

Positions	X (cm)	Y (cm)	Z (cm)
Microphone m_1_	280	213.2	112
Microphone m_2_	277.9	212.1	112
Microphone m_3_	276.8	210	112
Microphone m_4_	277.9	207.9	112
Microphone m_5_	280	206.8	112
Microphone m_6_	282.1	207.9	112
Microphone m_7_	283.2	210	112
Microphone m_8_	282.1	212.1	112
Speaker 1	115	327	183
Speaker 2	136	84	165
Speaker 3	461	245	174
Room dimensions	560	420	315

**Table 2:** The MAEE results (in cm) for the proposed TCDMA-AGGPM algorithm in comparison with HiGRID, SH-TMSBL, SF-MCA, and TF-MW-BNP-AHB methods on real and simulated data, for 2 simultaneous speakers and for reverberant (scenario 1), noisy (scenario 2), and noisy-reverberant (scenario 3) environments.

MAEE (cm)	HiGRID [19]		SH-TMSBL [21]		SF-MCA [24]		TF-MW-BNP-AHB [25]		Proposed TCDMA-AGGPM	
**Simulated Data**										
**Speaker**	**S1**	**S2**	**S1**	**S2**	**S1**	**S2**	**S1**	**S2**	**S1**	**S2**
**Scenario 1** **(Reverberant)**	57	52	45	51	48	43	36	38	32	35
**Scenario 2** **(Noisy)**	45	41	36	40	39	37	31	34	25	28
**Scenario 3** **(Noisy-Reverberant)**	74	68	61	67	64	59	47	52	42	45
**Real Data**										
**Speaker**	**S1**	**S2**	**S1**	**S2**	**S1**	**S2**	**S1**	**S2**	**S1**	**S2**
**Scenario 1** **(Reverberant)**	61	56	49	55	50	47	39	41	34	37
**Scenario 2** **(Noisy)**	47	44	39	43	40	41	32	36	30	33
**Scenario 3** **(Noisy-Reverberant)**	77	73	68	71	68	65	55	58	44	47

**Table 3:** The MAEE results (in cm) for the proposed TCDMA-AGGPM algorithm in comparison with HiGRID, SH-TMSBL, SF-MCA, and TF-MW-BNP-AHB methods on real and simulated data, for 3 simultaneous speakers and for reverberant (scenario 1), noisy (scenario 2), and noisy-reverberant (scenario 3) environments.

MAEE (cm)	HiGRID [19]			SH-TMSBL [21]			SF-MCA [24]			TF-MW-BNP-AHB [25]			Proposed TCDMA-AGGPM		
	**Simulated Data**														
**Speaker**	**S1**	**S2**	**S3**	**S1**	**S2**	**S3**	**S1**	**S2**	**S3**	**S1**	**S2**	**S3**	**S1**	**S2**	**S3**
**Scenario 1** **(Reverberant)**	48	53	51	44	47	48	41	45	43	33	34	37	27	30	31
**Scenario 2** **(Noisy)**	46	49	47	41	45	46	39	43	42	32	33	35	26	28	28
**Scenario 3** **(Noisy-Reverberant)**	71	74	77	68	72	70	62	69	65	51	55	54	41	45	46
	**Real Data**														
**Speaker**	**S1**	**S2**	**S3**	**S1**	**S2**	**S3**	**S1**	**S2**	**S3**	**S1**	**S2**	**S3**	**S1**	**S2**	**S3**
**Scenario 1** **(Reverberant)**	52	57	55	45	48	50	43	46	44	35	37	38	31	33	34
**Scenario 2** **(Noisy)**	49	53	51	44	46	49	41	45	40	37	40	43	30	32	31
**Scenario 3** **(Noisy-Reverberant)**	75	79	78	71	74	73	68	72	70	53	57	59	45	47	48

**Table 4:** The run-time (in seconds) comparison between the proposed TCDMA-AGGPM, HiGRID, SH-TMSBL, SF-MCA, and TF-MW-BNP-AHB methods for 2 and 3 simultaneous speakers on real data in noisy-reverberant environments.

Run-Time (s)	HiGRID [19]	SH-TMSBL [21]	SF-MCA [24]	TF-MW-BNP-AHB [25]	Proposed TCDMA-AGGPM
	**2 Simultaneous Speakers**				
**Scenario 1** **(Reverberant)**	627	530	384	443	245
**Scenario 2** **(Noisy)**	584	508	352	419	213
**Scenario 3** **(Noisy-Reverberant)**	665	567	401	468	259
	**3 Simultaneous Speakers**				
**Scenario 1** **(Reverberant)**	651	559	399	465	262
**Scenario 2** **(Noisy)**	632	526	374	457	248
**Scenario 3** **(Noisy-Reverberant)**	683	592	422	476	271

## Data Availability

Not applicable.

## Abbreviations

The following abbreviations are used in this manuscript:

ADMM	Alternative direction method of multipliers
AHB	Acoustical holography beamforming
AOA	Angle of arrival
BNP	Bayesian nonparametric
CC	Cross-correlation
CMA	Circular microphone array
DMA	Distributed microphone array
DNN	Deep neural networks
DOA	Direction of arrival
F-CRNN	Full-band recurrent neural networks
FIR	Finite impulse response
GCC	Generalized cross-correlation
GCC-PHAT	Generalized cross-correlation-phase transform
GCC-PHAT/ML	Generalized cross-correlation-phase transform/maximum likelihood
GEVD	Generalized eigenvalue decomposition
IFT	Inverse Fourier transform
IGMM	Infinite Gaussian mixture model
LMS	Least mean square
LTI	Linear time-invariant
MAEE	Mean absolute estimation error
ML	Maximum likelihood
MSE	Mean square error
MUSIC	Multiple signal classification
PDF	Power density function
PHAT	Phase transform
RIR	Room impulse response
RT_60_	Reverberation time
SD	Standard deviation
SF-MCA	Sound field morphological component analysis
SH	Spherical harmonic
SHC	Spherical harmonic domain
SH-TMSBL	Temporal extension of multiple response model of sparse Bayesian learning with spherical harmonic
SMIPL	Speech, music, and image processing laboratory
SNR	Signal-to-noise ratio
SRP	Steered response power
SRPD	Steered response power density
SRP-PHAT	Steered response power-phase transform
SSL	Sound source localization
TCDMA-AGGPM	T-shaped circular distributed microphone array-adaptive generalized eigenvalue decomposition, generalized cross-correlation-phase transform/maximum likelihood
TDOA	Time difference of arrival
TF	Time-frequency
TIMIT	Texas Instruments and Massachusetts Institute of Technology
UTEM	Universidad Tecnológica Metropolitana
VAD	Voice activity detection
W-DO	Windowed-disjoint orthogonality
WGN	White gaussian noise
WM	Mixture weight
